# Adherence of CAMHS Community Center, Winsford to the NICE Guidelines With Regards to Identification and Management in Depression in Children and Young People (NG134)

**DOI:** 10.1192/bjo.2022.457

**Published:** 2022-06-20

**Authors:** Sanjeet Kour

**Affiliations:** Cheshire and Wirral Partnership NHS Foundation Trust, Chester, United Kingdom

## Abstract

**Aims:**

To evaluate whether the practice in the generic CAMHS team Winsford is in line with the guidelines recommended by NICE in identification and management of depression in CYP. To formulate an action plan that might be needed for the recommendations that are not met currently.

**Methods:**

To collect the relevant information about the identification and management of depression in young people in our community center by following methods:

1. Review of online case notes, protocols, pathway descriptions, screening forms and proformas
2. Random review of the last 12 months of practice with random five cases studied per case manager regarding identification and management of depression in the children and young people at the center. To assess this, a proforma will be prepared from the guidelines relevant to the team members. This proforma will be sent to all the clinical workers of the team who will be required to fill it and return it to the lead author.

The population to be included will be all secondary school aged children residing in West Cheshire who are referred to and assessed and managed by the CAMHS community center, Winsford

**Results:**

I am working on this audit currently and will be obtaining the results in two months’ time and hopefully will be able to submit the audit poster well before the International Congress.

**Conclusion:**

This audit will help the team to assess how diligently they are following the recommended NICE guidelines for the identification and management of depression in children and young people and to make appropriate changes in the process to meet the guidelines that are not currently met.

